# Clozapine and anti-cancer agents: a systematic literature review of case reports

**DOI:** 10.1017/S1092852925100576

**Published:** 2025-09-24

**Authors:** Amity Honor, Joseph Parmenter, Rodney Marsh, Dan Siskind, Nicola Warren

**Affiliations:** 1 Medical School, The University of Queensland, Brisbane, QLD, Australia; 2 Tess Cramond Pain and Research Centre, Royal Brisbane and Women’s Hospital, Brisbane, QLD, Australia; 3 Mental Health Service, Royal Brisbane and Women’s Hospital, Brisbane, QLD, Australia; 4 Centre for Clinical Research, The University of Queensland, Brisbane, QLD, Australia; 5 Mental Health Service, Metro South Addiction and Mental Health, Brisbane, QLD, Australia

**Keywords:** Clozapine, cancer, chemotherapy, schizophrenia, psychosis

## Abstract

Clozapine is the gold standard for treatment-resistant schizophrenia. In the setting of malignancy with concurrent anti-cancer agent use, clozapine use may be of increased concern. Clozapine cessation holds its own risks. This study aims to systematically review all cases of concurrent pharmacotherapy with clozapine and anti-cancer agents and analyze the psychiatric and physical health outcomes. PubMed, EMBASE, CINAHL, and PsycINFO databases were searched from inception to February 2025. Descriptive statistics and narrative analysis of the included cases occurred. There were 53 cases of clozapine use with anti-cancer agents, with a male to female ratio of 1.7:1 and a mean age of 45.0 years. In 30 cases, clozapine was continued without interruption, and in additional 16 cases, clozapine was recommenced after a period of interruption. In cases with clozapine interruption or discontinuation, 90% noted significant deterioration in mental state despite alternative antipsychotic treatments. There were 34 cases of neutropenia, mostly (94%) in the setting of cytotoxic chemotherapy, with low rates of neutropenic complications. The successful continuation of clozapine with anti-cancer agents can occur, although risk-benefit analysis taking into account individual, clozapine, psychiatric, and physical health factors is required. Consideration of prophylactic neutropenia protective measures should form part of the discussion with the individual and their family.

## Introduction

Clozapine is the gold standard antipsychotic for individuals with treatment-resistant schizophrenia, offering superior efficacy in reducing psychotic symptoms, hospitalization, suicide, and all-cause mortality, as well as improving quality of life.^1^ ^-^^7^ Clozapine is, however, associated with serious potential adverse drug reactions (ARDs), including neutropenia and myocarditis at the time of clozapine commencement, as well as ongoing ADRs, including gastrointestinal hypomotility, cardiomyopathy, sedation, and sialorrhea.^8^ ^-^^11^ Many of these ongoing ADRs are influenced by clozapine plasma levels, with toxicity typically occurring from 600 to 1,000 ng/ml, resulting in seizures and other neurotoxic effects.^12^ ^,^^13^ It is with these factors in mind that the use of clozapine may be of particular concern during treatment with anti-cancer agents.

Anti-cancer agents include traditional cytotoxic chemotherapy and newer classes of medicines with various mechanisms of action, such as anti-tumor antibiotics, immunotherapies, alkylating, and hormonal agents. Myelosuppressive chemotherapy and subsequent neutropenia may be of particular concern with concurrent clozapine.^14^ Anti-cancer agents may also have gastrointestinal, cardiotoxic, or sedative effects, which may be additive to clozapine’s ADRs.^15^ ^,^^16^ Metabolism of clozapine may be affected by kidney or liver impairment, as well as any anti-cancer agent that impacts cytochrome P450 (CYP1A2) enzymes, increasing the risk of clozapine toxicity.^17^ ^,^^18^

Equally, there may also be risks of clozapine discontinuation. Clozapine cessation not only carries the risk of withdrawal-associated return of symptoms, but can also be associated with rebound cholinergic symptoms—such as gastrointestinal disturbance, delirium, and insomnia—as well as serotonergically driven agitation, diaphoresis, and neurological disturbance.^19^ New onset catatonia and “super-sensitivity psychosis” (rapid and severe onset of psychosis) have also been described with clozapine reduction and are thought to be more common with abrupt cessation.^20^ These potential withdrawal effects would likely have an additional impact on the individual’s cancer treatment, engagement with health services, interaction with family and/or caregivers, and quality of life.

Individuals with schizophrenia are thought to have an increased risk of some malignancies. The rates of lung and breast cancer, in particular, have been shown to be elevated, primarily thought to be associated with increased exposure to cancer risk factors such as tobacco and other substance use, obesity, and other health-related habits.^21^ ^,^^22^ Clozapine is thought to be associated with a small but statistically significant increase in hematological malignancy.^23^ Additionally, individuals with schizophrenia have an increased risk of mortality from cancer, possibly influenced by impaired health literacy, poor access to screening and treatment services, and increased physical health multimorbidity.^24^ ^-^^26^ Prompt decisions around pharmacotherapy are, therefore, vital.

A previous review focused on the myelosuppressive effect of chemotherapy and clozapine, reporting one episode of significant neutropenia out of 27 cases.^27^ Other potential interactions between clozapine and anti-cancer agents were not considered, and new agents recommended for cancer treatment were not examined. The aim of this systematic review was to describe the evidence obtained from case reports for co-administration of clozapine and cancer-related pharmacotherapy, and to synthesize the data regarding the positive and negative psychiatric and physical health outcomes.

## Methods

The Preferred Reporting Items for Systematic Reviews and Meta-Analysis (PRISMA) 2020 guideline was followed, and the protocol was pre-registered with the International Prospective Register of Ongoing Systematic Reviews (CRD 42025649070).^28^

The databases PubMed, EMBASE, CINAHL, and PsycINFO were searched from inception to February 2025. Search terms included (Clozapine OR Clozaril OR Clopine OR Zaponex) AND (“chemotherapy” OR “antineoplastic” OR “anti-neoplastic” OR “anti-cancer” OR “immunotherapy” OR [individual anti-neoplastic drug names]). The full list of search terms is included in the Supplementary Material. A comprehensive list of anti-cancer agents to include in the literature search was obtained from the digital Australian Medicines Handbook, Anticancer Drugs chapter.^29^ Screening of records’ titles and abstracts was conducted independently by 2 reviewers (A.H. and N.W.), resolving any conflicts with all authors following initial screening. Subsequent review of full texts by 2 reviewers (A.H. and N.W.) was conducted.

Included study types were case reports or case series, which described individuals prescribed clozapine for any indication and concurrently or within1 month, prescribed an anti-cancer agent for any indication. The time frame was chosen to include individuals where prescribed clozapine was ceased due to the commencement of an anti-cancer agent. Given the small numbers of identified cases in previous reviews, as well as the emerging cross-utility of the pharmacotherapy, the indication for anti-cancer agent use was kept broad. This was intended to capture instances where chemotherapeutic agents were prescribed for non-oncological conditions, such as inflammatory and autoimmune diseases. Anti-rejection medicines, including calcineurin inhibitors, immunosuppressant antibodies, and mammalian target of rapamycin inhibitors, were also included due to the immunosuppressive and/or cytotoxic effects of these agents, their use in other cancer treatments such as hematopoietic stem cell transplants, and the overlap in drug classes.

All ages and languages, translatable to English, were included. Individual case reports were extracted from studies containing case series or multiple case reports based on relevance. Again, in an attempt to capture the greatest scope of information, poster and presentation abstracts were included, although studies lacking individual case data or opinion pieces were excluded. Cases of clozapine-induced agranulocytosis, without a proximal anti-cancer agent, were excluded. Cases with historical clozapine, but not current, in the setting of anti-cancer agent commencement, were also excluded.

Data variables extracted included: study details (publication year and country); patient demographics (age and sex); patient psychiatric history (functioning prior to and post clozapine, decision-making capacity, diagnoses, and medications); clozapine details (indication, treatment duration, dose, trough levels, treatment details during anti-cancer agent commencement, and side effects); anti-cancer agent details (indication, protocol used, and treatment duration); rationale for clozapine decision and stakeholders involved; hematological monitoring details; adverse events (details, attributed cause, intervention, and complications); and psychiatric and physical health outcomes. The use of granulocyte-colony stimulating factor (G-CSF) or other pharmacologic supportive therapies for side effect and toxicity management was recorded. Missing data were noted.

Descriptive statistics and narrative analysis were conducted as initial scoping suggested insufficient studies to use meta-analysis. Student’s *t*-test or chi-square test was used to compare cases developing neutropenia and those that did not. Narrative analysis was conducted according to the synthesis without meta-analysis reporting guidelines.^30^ Study quality assessment was conducted using the Joanna Briggs Institute (JBI) critical appraisal checklist for case reports.^31^

## Results

**Figure 1.**
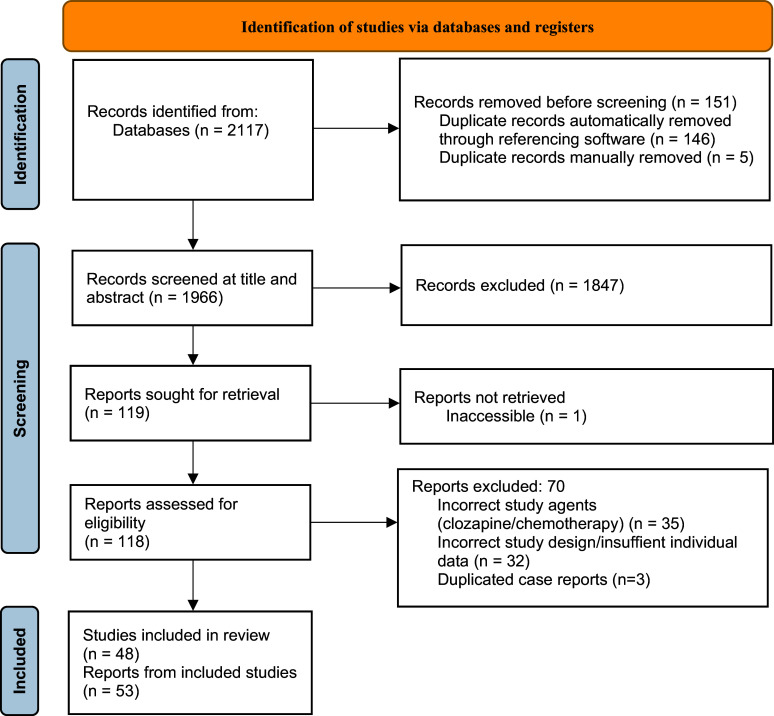
PRISMA 2020 flow diagram for clozapine and anti-cancer agents.

Of the 1,966 non-duplicated articles screened at the title, abstract, and full-text levels, there were 48 studies, with 53 individual cases reported from 1993 to 2024, across 16 different countries (Figure 1, Table 1, and Supplementary Material).^32^ ^-^^79^ The average JBI quality assessment score was 4.7 out of 8 (SD 1.8), with 12 studies (23.1%) scoring below 4/8 (Supplementary Material).

**Table 1.** Individual Studies of Clozapine and Anti-Cancer Agents

First author	Year	Country	Age	Sex	Indication for clozapine	Indication for ACA
Avnon	1993	Israel	46	F	SCZ	Uterine endometrial carcinoma, type II B papillary serous ovary adenoma
Bampton	2018	Australia	44	M	SCZ	Ileocaecal Crohn’s disease
Bareggi	2002	Italy	37	M	SCZ	Undifferentiated nasopharyngeal carcinoma
Barreto	2015	USA	55	M	SCZ-A	Burkitt lymphoma
Breitschwerdt	2019	USA	14	M	SCZ	Autoimmune encephalitis
Burlingham	2016	England	63	F	SCZ	Primary amyloidosis confined to kidneys
Campbell	2020	England	48	M	SCZ	B-cell lymphoma of the small bowel
Ceran	2024	Turkey	47	F	SCZ	“Breast and ovarian cancers”
Chamberlain	2015	England	n/s	F	SCZ	Hodgkin lymphoma
Chang	2015	Taiwan	22	M	SCZ	Acute myeloid leukemia
Chen	2020	Australia	34	M	SCZ	Proliferative glomerulonephritis
Conroy	2018	USA	25	F	SCZ	Anti-NMDA encephalitis
Cunningham	2014	USA	56	M	SCZ	Lung squamous cell carcinoma with brain metastases
De Berardis	2013	Italy	37	M	SCZ	Behcet’s disease
Deodhar	2014	India	39	M	SCZ	Cancer of the tongue
Doǧan	2023	India	43	M	SCZ	Hodgkin lymphoma
			48	M	SCZ-A	Hodgkin lymphoma
Francis	2019	Malaysia	47	F	SCZ	Adenocarcinoma of breast
Frieri	2008	Italy	44	M	SCZ	Follicular non-Hodgkin lymphoma
Goulet	2008	Canada	51	M	SCZ	Small cell lung carcinoma with hepatic metastases
Haut	1995	Scotland	41	M	SCZ	High grade non-Hodgkin lymphoma, duodenum mass
Hundertmark	2001	Australia	n/s	n/s	SCZ	Diffuse large B cell lymphoma
Kilincaslan	2017	Turkey	16	M	Behavior	Tuberous Sclerosis Complex
Kolli	2013	USA	46	M	SCZ	Diffuse large B-cell lymphoma, CNS involvement
Kutzke	2021	USA	42	M	SCZ	Refractory diffuse large B-cell lymphoma
Langebrake	2022	Germany	52	F	SCZ	Primary myelofibrosis
Liu	2010	USA	n/s	F	SCZ-A	Chronic lymphocytic leukemia
Mamtani	2024	India	33	F	BPAD I	Multiple sclerosis
McKenna	1994	USA	49	M	SCZ	Small cell lung cancer
Monga	2015	USA	70	M	SCZ	Squamous cell carcinoma of the distal esophagus
Moraes de Brito	2023	Brazil	44	F	SCZ	Diffuse large B-cell lymphoma
Munshi	2013	Canada	58	M	SCZ	Large B-cell lymphoma
O’Leary	2018	Ireland	55	F	BPD	Vulval carcinoma
Overbeeke	2016	Netherlands	45	M	SCZ	Follicular non-Hodgkin lymphoma
Padda	2022	USA	59	M	SCZ	Liver transplant, non-alcoholic steatohepatitis
Pakhre	2016	India	38	F	BPAD I	Ovarian carcinoma (papillary adenocarcinoma)
Riddle	2017	USA	60	F	SCZ	Rectal carcinoma
Rosenberg	2007	USA	39	M	BPAD I	Hodgkin lymphoma nodular sclerosing type
Rosenstock	2004	USA	46	F	SCZ	Breast cancer
Sankaranarayanan	2013	Australia	53	M	SCZ	Seminoma
			41	F	SCZ-A	Breast cancer
			32	F	SCZ	Colon cancer
Sharma	2023	Nepal	54	M	SCZ	Extra-pulmonary sarcoidosis (ocular and cutaneous)
Taylor	2024	England	60s	M	n/s	Myeloma
			50s	M	n/s	Lymphoma
			50s	M	n/s	“Cancer” (unspecified)
Tellez	2019	USA	78	F	SCZ-A	Endometrial adenocarcinoma with local metastases
Usta	2014	Turkey	53	M	SCZ	Chronic lymphocytic leukemia and lymphoma
Van Gool	2008	Netherlands	37	F	SCZ	Cervical cancer
Wesson	1996	England	n/s	M	SCZ	Testicular teratoma with pulmonary metastases
Wright	2022	USA	30	M	SCZ	B-cell acute lymphocytic leukemia
Yoong	2023	Australia	56	M	SCZ	Merkel cell carcinoma with bone and hepatic metastases
Zhang et al.	2023	Australia	45	F	SCZ	Colon cancer

Abbreviations: ACA, anti-cancer agent; BPAD, bipolar affective disorder; BPD, borderline personality disorder; CNS, central nervous system; F, female; GvHD, graft versus host disease; HSCT, haemopoietic stem cell transplant; M, male; n/s, not stated; NMDA, N-methyl-D-aspartate; SCZ, schizophrenia; SCZ-A, schizoaffective disorder.

### Description of cases

The mean age of cases was 45.0 years (SD 12.6), ranging from 14 to 78 years, with 3 cases reporting an age range only and 4 cases not reporting on age. The male-to-female ratio was 1.7:1, with 1 case not stated.

Schizophrenia was the most common indication for clozapine (40 cases, 75.5%), with other indications, including schizoaffective disorder (5 cases, 9.4%), bipolar affective disorder (3 cases, 5.7%), and borderline personality disorder (1 case, 1.9%). In 1 case, clozapine was used for the treatment of aggression and emotional dysregulation in the context of Tuberous Sclerosis. The indication for clozapine was not stated in 3 cases. In the 37 cases that reported on clozapine dosing, the dose ranged from 37.5 to 900.0 mg daily (mean 417.0 mg, SD 197.0). There were 46 cases reporting the duration of clozapine treatment, ranging between 3 months and 25 years (mean 11.0 years, SD 7.0) prior to anti-cancer agent commencement, with an additional 3 cases reporting clozapine commencement after the initiation of anti-cancer agent, and 4 cases not reporting on timing. Psychotropics, in addition to clozapine, were used in 22 cases (41.5%), which were most frequently antidepressants (10 cases), concurrent antipsychotics (8 cases), mood stabilizers (5 cases), and benzodiazepines (3 cases).

Malignancy was the most common indication for anti-cancer agent commencement, reported in 43 cases (81.1%); of these, 22 cases were non-hematological, 20 cases were hematological, and 1 case was unspecified. Non-oncological indications for anti-cancer agent use included 2 cases of autoimmune encephalitis and 1 case each of amyloidosis, Crohn’s disease, sarcoidosis, Behçet’s disease, tuberous sclerosis, multiple sclerosis, proliferative glomerulonephritis, and liver transplant.

The anti-cancer agents included 30 medicines from 15 different drug classes (Supplementary Material). In 35 cases, only traditional cytotoxic chemotherapy agents (66.0%) were used; combined chemotherapy and immunotherapy agents were used in 9 cases (17.0%); and 8 cases used immunotherapy only (15.1%). Multiple concomitant anti-cancer agents (irrespective of drug class) were used in 29 cases (54.7%).

### Changes to clozapine

There were 22 cases (41.5%) reporting no change to clozapine dosing during treatment with an anti-cancer agent. Physical health outcomes were positive in all but 3 cases: McKenna et al.^55^ reported a case in which a patient declined continuation of chemotherapy after 1 cycle, not attributed to lack of capacity or psychiatric diagnosis; Taylor et al.^67^ described a case with rapid neoplastic disease progression, resulting in death; Moraes de Brito et al.^57^ reported a case with relapse of lymphoma 18 months after remission and speculated that clozapine may have predisposed recurrence.

In 5 cases (9.4%), continued clozapine prescription with an alteration to the dose was reported. Overbeeke et al.^60^ reported on increased clozapine dose prescribed in response to the psychiatric side effects of high-dose corticosteroids for non-Hodgkin’s lymphoma, later reducing clozapine dose to avoid toxic clozapine levels in the context of a concomitant infection. Similarly, Padda et al.^61^ described an increased clozapine dose due to corticosteroid-related psychotic and affective symptoms in the context of renal transplant. Conroy et al.^43^ reported a clozapine dose reduction due to mental state improvement with immunotherapy for autoimmune encephalitis. Similarly, De Berardis et al.^45^ noted a decrease in clozapine dose due to mental state stability following azathioprine use for Bechet’s disease. In the case reported by Deodhar et al.^46^, clozapine reduction was required, initially to mitigate dose-dependent sialorrhea, and subsequently in the anticipation of a potential synergistic effect on chemotherapy.

There were 16 cases (30.2%) that reported interrupted clozapine prescription with later recommencement. The duration of interruption, reported in 11 cases, ranged from 2 days to approximately 5 months. The most common reason for interruption in therapy (10 cases, 18.9%) was due to the onset of neutropenia during treatment with the anti-cancer agent. Additionally, in 1 case, Hundertmark et al.^73^ reported neutropenia due to diffuse large B cell lymphoma, which initially necessitated clozapine cessation, but in which later clozapine was recommenced due to mental state deterioration unresponsive to other antipsychotics. Three cases were interrupted due to the oncology team’s concern for the potential risk of neutropenia, without such occurring. Doǧan et al.^47^ cited the family’s preference as the reason for interrupting clozapine; Frieri et al.^48^ did not provide an explanation.

There were 7 cases (13.2%) where clozapine was discontinued. Beritschwerdt et al.^37^ described a complex case where a 14-year-old was initially diagnosed with schizophrenia and treated with clozapine, later found to have autoimmune encephalitis and neurobartonellosis, improving with rituximab and no longer requiring any antipsychotic. Chang et al.^41^ reported on clozapine cessation at the time of anti-cancer agent commencement, citing the risk of myelosuppression. Three cases reported clozapine cessation during anti-cancer agent use due to neutropenia, and 2 cases reported clozapine cessation 4 and 10 weeks after anti-cancer agent treatment due to neutropenia.

In 3 cases (5.7%), clozapine was started during existing treatment with an anti-cancer agent. Bampton et al.^34^ described an individual with schizophrenia and Crohn’s disease treated with azathioprine, who was successfully commenced on clozapine, resulting in psychotic symptom remission. Sharma et al.^66^ reported on the commencement of clozapine for schizophrenia in an individual with sarcoidosis on methotrexate and corticosteroids, resolving psychotic symptoms after multiple previous unsuccessful antipsychotic trials. Kilincaslan et al.^50^ noted a 16-year-old on Everolimus for Tuberous Sclerosis, whose aggressive behavior was reported to resolve after clozapine commencement.

### Psychiatric outcomes

In the 30 cases (56.7%) where clozapine was continued unchanged, continued with dose adjustment, or commenced during anti-cancer agent use, no significant mental state deterioration was reported, with all cases noting positive psychiatric outcomes. Of the 16 cases where clozapine was interrupted, all reported significant deterioration in mental state despite alternative antipsychotic use, particularly with longer periods of interruption. Of those that reported longer-term psychiatric outcomes (12 cases), all noted improved or resolved mental state with clozapine recommencement. The impact of psychiatric deterioration was significant for many; for example, Campbell et al.^38^ reported a case where clozapine was interrupted due to neutropenia for a total of 4 months, with a significant deterioration in mental state resulting in a protracted involuntary mental health admission, interruption of chemotherapy treatment, failure of 2 subsequent alternative antipsychotic trials, and ultimately being recommenced on a higher dose of clozapine. Of the 6 cases where clozapine was discontinued (excluding the case with the resolution of underlying pathology), 4 cases noted significant deterioration in mental state despite alternative antipsychotics, and 2 cases described stabilized mental state, on olanzapine^76^ and ziprasidone.^36^

### Neutropenia

Neutropenia arose in 34 cases (64.2%), of which 16 had an underlying hematological malignancy, 18 reported a non-hematological malignancy, and 1 reported a liver transplant. The majority of these cases were on cytotoxic chemotherapy (32 cases), but there was no difference in the use of multiple anti-cancer agents compared to a single agent (65.6% vs. 40.0%, *p* = 0.07). The case report authors attributed the neutropenia to the anticancer agent in 31 cases and clozapine in 3 cases. The mean age of cases with neutropenia was 48.8 years (SD 9.6), which was significantly older than cases that did not develop neutropenia (39.8 years, SD 14.2, *p* = 0.012). There was 1 death related to neutropenia^67^, and 7 cases reported other neutropenia-related complications, including 4 cases of sepsis, 2 cases of febrile neutropenia, and 1 case of bronchopneumonia. G-CSF was administered in 20 cases, with continued (12 cases) or interrupted (5 cases) clozapine in the majority. In 3 cases, neutropenia continued despite G-CSF and resulted in the cessation of clozapine. No case used prophylactic antibiotics.

### Other adverse effects

There were a few other adverse effects noted during concurrent clozapine and anti-cancer agent treatment. Zhang et al.^72^ reported constipation with fecal impaction and subsequent bowel perforation in the setting of bowel cancer. Kutzke et al.^52^ reported a case with deep vein thrombosis and pulmonary embolism, cytokine release syndrome, and neurotoxicity in the setting of diffuse large B cell lymphoma and multiple anti-cancer agents. Francis et al.^74^ described a case with hypotension limiting clozapine up-titration, and as noted earlier, Deodhar et al.^46^ reported significant sialorrhea requiring clozapine dose reduction. No case reported clozapine toxicity or cardiotoxicity effects. There were 4 cases reporting pre-existing metabolic syndrome that did not change during anti-cancer agent treatment, and there were no new cases developing such.

### Stakeholder involvement and individualized management planning

Individualized stakeholder involvement in decision-making was reported in 31 cases (58.5%), and in 27 cases (51.9%), an individual risk-benefit analysis to determining clozapine management was noted. Of these, 13 cases documented involving the patient in decision-making; 10 included the patient’s family or nominated support person; 1 liaised with a substitute maker; and multi-specialty or multi-disciplinary consultation was reported in 21 cases.

## Discussion

This is the first systematic review of clozapine with the concurrent use of a wide range of anti-cancer agents, reporting on 53 cases where the majority (86.8%) continued or recommenced clozapine. In cases with a primary mental illness where clozapine was interrupted or discontinued, all but 2 cases reported significant deterioration in mental state despite alternative antipsychotics. Neutropenia occurred in over half the cases, many reporting the use of G-CSF to support ongoing clozapine use. Non-hematological ADRs of clozapine were less frequently reported, including no reports of clozapine toxicity.

Neutropenia is a key concern with myelosuppressive anti-cancer agents due to the increased mortality and morbidity from both neutropenic complications, as well as from the underlying cancer if anti-cancer agents need to be reduced or ceased.^80^ Incidence of neutropenia from anti-cancer agents is estimated between 1.6 and 15.4 cases per million per year, with febrile neutropenia or neutropenia resulting in hospitalization occurring in 7.8 per 1,000 individuals with cancer.^81^ ^-^^83^ Certain classes of anti-cancer agents are associated with a greater risk, such as anthracyclines, taxanes, alkylators, and topoisomerase inhibitors, seen most frequently in the initial cycle of chemotherapy.^83^ ^,^^84^ This may be further increased when multiple anti-cancer agents are used and with other risk factors such as older age, lower body mass, renal and liver dysfunction, and bone marrow involvement.^80^ ^,^^84^ Although there was a high rate of neutropenia seen in this review’s cases, most were noted on routine testing, as opposed to presenting with febrile neutropenia or other complications. Publication bias may also distort the rate of neutropenia. Large epidemiological studies have demonstrated the peak of clozapine associated neutropenia to be at 9 weeks post commencement, falling at around 18 weeks to an incidence of 0.001% by 2 years, and thus for those on stable long-term clozapine, there may not be an additive neutropenic risk.^8^

Evidence-based guidelines from America and Europe now include the recommendation for G-CSF as prophylaxis for neutropenia in those at “higher risk” while receiving myelosuppressive anti-cancer agents.^80^ Higher risk is determined to be when anti-cancer agents are associated with neutropenia in over 20%, or over 10% when another risk factor is identified. G-CSF has been shown to be a safe, effective, and well-tolerated treatment for clozapine associated neutropenia that can occur on initial commencement.^85^ Consideration of G-CSF or antibiotic prophylaxis should form part of the risk-benefit discussion.

Other clozapine and anti-cancer agent interactions were not frequently reported in the cases of this review. Potentially, symptoms such as sedation, constipation, and urinary incontinence were attributed to the underlying physical health disorder. Clozapine levels were infrequently assessed despite the increased risk of toxicity with inflammation and infection.^18^ Altered symptom awareness and communication challenges may have reduced the identification of these concerns.^86^ It is also possible that clozapine ADRs may have provided benefit in the setting of cancer and anti-cancer agent use. For example, appetite stimulation may counteract nausea, reduced gastric motility may be beneficial in the setting of increased motility, and hypersalivation may assist with xerostomia. There is also an emerging understanding of the immunomodulatory effect of clozapine, which may also contribute.^32^

If the decision is to stop clozapine, there is limited evidence to guide on tolerability, efficacy, and pharmacological interactions of alternative psychosis management in this setting. Antipsychotic polypharmacy, typical antipsychotics, and electroconvulsive therapy, traditionally recommended when clozapine is unsuitable, may not be ideal options.^87^ ^,^^88^ Individuals undergoing active treatment for cancer may have difficulty engaging in psychological therapies for adjunctive psychosis treatment. The experience of decompensation, the impact on the person, their self-perception, and their community, the risk of suicide, violence, and reputational damage need to be examined.

There are inherent limitations with using data from case reports, with publication bias thought to particularly impact the reporting of negative outcomes. However, it is important to synthesize this information, as concurrent clozapine and anti-cancer agent use in practice may be limited by the assumption of complications as opposed to evidence for such. In an effort to report on the most complete set of anti-cancer agent and clozapine cases, there were 10 non-malignancy cases included in this review, which potentially have different symptom and risk profiles. The total number of cases reported here was still low, and it is vital that clinical decision-making around pharmacotherapy choice be an individualized process. This is especially important when considering the heterogeneity of the clozapine dosing, anti-cancer agent choice, and medical and psychiatric histories within these cases. It should also be appreciated that data are reliant on what is reported within the cases. Just over a quarter of the studies were non-psychiatrically focused (no psychiatrist authors, published in a non-psychiatric journal), which may have impacted the reporting of psychiatric outcomes. Similarly, for those with a psychiatric focus and reporting of cancer-based outcomes. Future studies would benefit from detailed documentation on pharmacological, psychiatric, and physical health parameters.

Successful continuation of clozapine with anti-cancer agents may occur, especially if the individual has been on clozapine for some time with a stable dose. Treatment planning should include consideration of pre-intervention probability of complications from anti-cancer agents based on individual assessment, with consideration of prophylactic myelosupportive agents. Clinical decisions should involve all stakeholders and recognition that outcomes will be improved with clear risk-benefit analysis, taking into account the individual’s psychiatric and physical needs, in addition to preferences.

## Supporting information

Honor et al. supplementary material Honor et al. supplementary material

## Data Availability

The authors confirm that the data supporting the findings of this study are available within this article and its supplementary material.

## Supplementary material

The supplementary material for this article can be found at http://doi.org/10.1017/S1092852925100576.
