# Supplementary material for: Clozapine and anti-cancer agents: a systematic literature review of case reports
Source: CNS Spectr. 2025 Sep 24;30(1):e76. doi: 10.1017/S1092852925100576 (PMC13064762; doi:10.1017/S1092852925100576)
Supplement: Honor et al. supplementary material [file S1092852925100576sup001.docx]

**Appendix A: Search Terms**

(Clozapine OR Clozaril OR Clopine OR Zaponex) AND (“chemotherapy” OR “antineoplastic” OR “anti-neoplastic” OR “anti neoplastic” OR “immunotherapy” OR Bendamustine OR Busulfan OR Carmustine OR Chlorambucil OR Cyclophosphamide OR Dacarbazine OR Ifosfamide OR Lomustine OR Melphalan OR Procarbazine OR Temozolomide OR Thiotepa OR Daunorubicin OR Doxorubicin OR Epirubicin OR Idarubicin OR Mitozantrone OR “Brentuximab vedotin” OR “Enfortumab vedotin” OR “Gemtuzumab ozogamicin” OR “Inotuzumab ozogamicin” OR “Sacituzumab govitecan” OR “Trastuzumab deruxtecan” OR “Trastuzumab emtansine” OR Azacitidine OR Capecitabine OR Cladribine OR Clofarabine OR Cytarabine OR Decitabine OR cedazuridine OR Fludarabine OR Fluorouracil OR Gemcitabine OR Hydroxycarbamide OR Mercaptopurine OR Methotrexate OR Pemetrexed OR Raltitrexed OR Tioguanine OR Trifluridine OR Tipiracil OR Abemaciclib OR Acalabrutinib OR Afatinib OR Alectinib OR Asciminib OR Axitinib OR Binimetinib OR Brigatinib OR Cabozantinib OR Ceritinib OR Cobimetinib OR Crizotinib OR Dabrafenib OR Dasatinib OR Encorafenib OR Entrectinib OR Erlotinib OR Gefitinib OR Gilteritinib OR Ibrutinib OR Idelalisib OR Imatinib OR Lapatinib OR Larotrectinib OR Lenvatinib OR Lorlatinib OR Midostaurin OR Nilotinib OR Nintedanib OR Osimertinib OR Palbociclib OR Pazopanib OR Ponatinib OR Regorafenib OR Ribociclib OR Ripretinib OR Ruxolitinib OR Sorafenib OR Sunitinib OR Tepotinib OR Trametinib OR Vemurafenib OR Zanubrutinib OR Carboplatin OR Cisplatin OR Oxaliplatin OR Bortezomib OR Carfilzomib OR Cabazitaxel OR Docetaxel OR Paclitaxel OR Lenalidomide OR Pomalidomide OR Thalidomide OR Irinotecan OR Topotecan OR “Vinca alkaloids” OR Vinblastine OR Vincristine OR Vinorelbine OR Anagrelide OR Arsenic trioxide OR BCG OR Bleomycin OR Dactinomycin OR Eribulin OR Etoposide OR Everolimus OR Mitomycin OR Niraparib OR Olaparib OR Pegaspargase OR Romidepsin OR Selinexor OR Sonidegib OR Trabectedin OR Tretinoin OR Venetoclax OR Vismodegib OR Vorinostat OR Alemtuzumab OR Atezolizumab OR Avelumab OR Bevacizumab OR Blinatumomab OR Cemiplimab OR Cetuximab OR Daratumumab OR Durvalumab OR Elotuzumab OR Ipilimumab OR Nivolumab OR Obinutuzumab OR Panitumumab OR Pembrolizumab OR Pertuzumab OR Rituximab OR Siltuximab OR Trastuzumab OR Amifostine OR “Calcium folinate” OR Mesna OR Palifermin OR Plerixafor OR Rasburicase OR Filgrastim OR Lipegfilgrastim OR Pegfilgrastim)

**Appendix B: Reason for Exclusion at Full Text**

| **Author/Year** | **Reason for Exclusion at Full Text** |
| --- | --- |
| Ali 2014 | No clozapine/Anti-cancer agent |
| Andre 2020 | No clozapine/Anti-cancer agent |
| Andrès 2017 | No clozapine/Anti-cancer agent |
| Augustin 2021 | No/insufficient individual data |
| Béchard 2021 | No clozapine/Anti-cancer agent |
| Bejerot 2023 | No clozapine/Anti-cancer agent |
| Boyle 2022 | No clozapine/Anti-cancer agent |
| Chrétien 2021 | No/insufficient individual data |
| Chrétien 2021 | No/insufficient individual data |
| Conchinha 2022 | No clozapine/Anti-cancer agent |
| Damjanović 2006 | No/insufficient individual data |
| Daniel 2016 | No/insufficient individual data |
| DaPonte 2014 | No/insufficient individual data |
| Fabrikant 2015 | No/insufficient individual data |
| Fernando 2017 | No clozapine/Anti-cancer agent |
| Fina 2022 | No/insufficient individual data |
| Fisch 2004 | No clozapine/Anti-cancer agent |
| Fond 2012 | No/insufficient individual data |
| Frantz 2005 | No/insufficient individual data |
| Friedman 2022 | No clozapine/Anti-cancer agent |
| Gavillet 2012 | No clozapine/Anti-cancer agent |
| Geibig 1993 | No clozapine/Anti-cancer agent |
| Gon 2016 | No clozapine/Anti-cancer agent |
| Grainger 2019 | No/insufficient individual data |
| Gupta 2017 | No/insufficient individual data |
| Hauvillier 2012 | No/insufficient individual data |
| He 2018 | No/insufficient individual data |
| Hoefnagel 2005 | No/insufficient individual data |
| Howard 2010 | No/insufficient individual data |
| Howell 2004 | No/insufficient individual data |
| Huang 2015 | No clozapine/Anti-cancer agent |
| Hwang 2012 | No/insufficient individual data |
| Javelot 2012 | No/insufficient individual data |
| Jesien-Lewandowicz 2010 | No clozapine/Anti-cancer agent |
| Kalash 1998 | No/insufficient individual data |
| Kayipmaz 2018 | Duplicated case |
| Kelleher 2020 | No clozapine/Anti-cancer agent |
| Khan 2013 | No clozapine/Anti-cancer agent |
| Kosek 2024 | No/insufficient individual data |
| Lee 2022 | No/insufficient individual data |
| Leung 2017 | No/insufficient individual data |
| Lewey 2022 | No clozapine/Anti-cancer agent |
| Lim 2006 | No clozapine/Anti-cancer agent |
| Lopez 2010 | No/insufficient individual data |
| Mancano 2018 | No clozapine/Anti-cancer agent |
| McKinnon 2000 | No/insufficient individual data |
| Meyer 2015 | No clozapine/Anti-cancer agent |
| Montastruc 2010 | No clozapine/Anti-cancer agent |
| O'Neill 2021 | No clozapine/Anti-cancer agent |
| Petit 1995 | No clozapine/Anti-cancer agent |
| Prely 2022 | No/insufficient individual data |
| Ramai 2022 | No clozapine/Anti-cancer agent |
| Reich 2009 | No clozapine/Anti-cancer agent |
| Rivers 2018 | No/insufficient individual data |
| Saylor 2011 | No/insufficient individual data |
| Sharma 2010 | No/insufficient individual data |
| Singh 2022 | No clozapine/Anti-cancer agent |
| Steriade 2014 | No clozapine/Anti-cancer agent |
| Strandell 2008 | No/insufficient individual data |
| Sulentic 2018 | No clozapine/Anti-cancer agent |
| Tzouma 2015 | No/insufficient individual data |
| vanderHeide 2024 | No clozapine/Anti-cancer agent |
| Vanelle 1993 | No clozapine/Anti-cancer agent |
| Voulgari 2015 | No clozapine/Anti-cancer agent |
| Wikerholmen 2022 | No clozapine/Anti-cancer agent |
| Wright 2021 | Duplicated case |
| Yap 2011 | No/insufficient individual data |
| Utsa 2013 | Duplicated case |
| Zandi 2014 | No clozapine/Anti-cancer agent |
| Zhang 2011 | No clozapine/Anti-cancer agent |

**Appendix C: Quality Assessment of Included Studies**

| **First Author** | **Year** | **1** | **2** | **3** | **4** | **5** | **6** | **7** | **8** |
| --- | --- | --- | --- | --- | --- | --- | --- | --- | --- |
| Avnon et al | 1993 | 1 | 1 | 1 | 0 | 0 | 0 | 0 | 1 |
| Bampton et al | 2018 | 1 | 0 | 0 | 1 | 1 | 1 | 1 | 1 |
| Bareggi et al | 2002 | 1 | 0 | 0 | 1 | 1 | 1 | 1 | 1 |
| Barreto et al | 2015 | 1 | 0 | 1 | 1 | 1 | 0 | 1 | 1 |
| Breitschwerdt et al | 2019 | 1 | 1 | 1 | 1 | 1 | 1 | 1 | 1 |
| Burlingham et al | 2016 | 1 | 0 | 0 | 1 | 1 | 1 | 1 | 1 |
| Campbell et al | 2020 | 1 | 0 | 0 | 0 | 1 | 1 | 1 | 1 |
| Ceran et al | 2024 | 1 | 0 | 0 | 0 | 1 | 1 | 1 | 1 |
| Chamberlain et al | 2015 | 0 | 0 | 0 | 0 | 1 | 0 | 0 | 1 |
| Chang et al | 2015 | 1 | 1 | 0 | 1 | 0 | 1 | 1 | 1 |
| Chen et al | 2020 | 1 | 0 | 0 | 1 | 1 | 0 | 0 | 0 |
| Conroy et al | 2018 | 1 | 0 | 0 | 1 | 1 | 1 | 1 | 1 |
| Cunningham et al | 2014 | 1 | 1 | 1 | 1 | 0 | 0 | 0 | 1 |
| De Berardis et al | 2013 | 1 | 0 | 1 | 1 | 1 | 1 | 1 | 1 |
| Deodhar et al | 2014 | 1 | 0 | 0 | 1 | 1 | 1 | 1 | 1 |
| Doǧan et al | 2023 | 1 | 0 | 0 | 0 | 1 | 0 | 1 | 1 |
| Francis et al | 2019 | 1 | 0 | 0 | 0 | 1 | 1 | 0 | 0 |
| Frieri et al | 2008 | 1 | 1 | 0 | 0 | 1 | 1 | 1 | 1 |
| Goulet et al | 2008 | 1 | 0 | 1 | 1 | 1 | 1 | 1 | 1 |
| Haut | 1995 | 1 | 1 | 1 | 0 | 1 | 1 | 1 | 1 |
| Hundertmark | 2001 | 0 | 0 | 1 | 0 | 1 | 1 | 1 | 1 |
| Kilincaslan et al | 2017 | 1 | 0 | 0 | 0 | 0 | 0 | 0 | 0 |
| Kolli et al | 2013 | 1 | 0 | 1 | 0 | 0 | 0 | 1 | 1 |
| Kutzke et al | 2021 | 1 | 1 | 1 | 1 | 1 | 1 | 1 | 1 |
| Langebrake et al | 2022 | 1 | 0 | 0 | 0 | 1 | 0 | 0 | 1 |
|  |  | 1 | 0 | 0 | 0 | 1 | 0 | 0 | 1 |
| Liu et al | 2010 | 0 | 1 | 1 | 0 | 1 | 1 | 1 | 1 |
| Mamtani et al | 2024 | 1 | 0 | 0 | 0 | 0 | 0 | 0 | 1 |
| McKenna et al | 1994 | 1 | 0 | 0 | 0 | 0 | 0 | 0 | 1 |
| Monga et al | 2015 | 1 | 1 | 0 | 0 | 1 | 0 | 1 | 1 |
| Moraes de Brito et al | 2023 | 1 | 0 | 1 | 1 | 1 | 0 | 0 | 1 |
| Munshi et al | 2013 | 1 | 0 | 0 | 0 | 1 | 1 | 1 | 1 |
| O'Leary et al | 2018 | 1 | 0 | 0 | 0 | 0 | 1 | 1 | 1 |
| Overbeeke et al | 2016 | 1 | 0 | 0 | 0 | 1 | 0 | 1 | 1 |
| Padda et al | 2022 | 1 | 0 | 0 | 0 | 1 | 0 | 1 | 1 |
| Pakhre et al | 2016 | 1 | 0 | 0 | 0 | 1 | 1 | 1 | 1 |
| Riddle et al | 2017 | 1 | 0 | 0 | 0 | 0 | 1 | 1 | 1 |
| Rosenberg et al | 2007 | 1 | 0 | 0 | 0 | 0 | 1 | 1 | 1 |
| Rosenstock | 2004 | 1 | 0 | 0 | 0 | 1 | 0 | 1 | 1 |
| Sankaranarayanan et al | 2013 | 1 | 0 | 0 | 0 | 1 | 1 | 0 | 1 |
|  |  | 1 | 0 | 0 | 0 | 1 | 0 | 1 | 1 |
|  |  | 1 | 0 | 0 | 0 | 0 | 0 | 1 | 1 |
| Sharma et al | 2023 | 1 | 0 | 0 | 1 | 1 | 0 | 1 | 1 |
| Taylor et al | 2024 | 0 | 0 | 0 | 0 | 0 | 0 | 0 | 1 |
|  |  | 0 | 0 | 0 | 0 | 0 | 0 | 0 | 1 |
|  |  | 0 | 0 | 0 | 0 | 0 | 0 | 0 | 1 |
| Tellez et al | 2019 | 1 | 0 | 0 | 0 | 1 | 1 | 1 | 1 |
| Usta et al | 2014 | 1 | 1 | 1 | 0 | 1 | 1 | 1 | 1 |
| Van Gool et al | 2008 | 1 | 0 | 1 | 0 | 1 | 1 | 1 | 1 |
| Wesson et al | 1996 | 0 | 1 | 1 | 1 | 1 | 1 | 1 | 1 |
| Wright et al | 2022 | 1 | 1 | 1 | 1 | 1 | 1 | 1 | 1 |
| Yoong | 2023 | 1 | 0 | 1 | 1 | 0 | 0 | 1 | 1 |
| Zhang et al | 2023 | 1 | 0 | 1 | 0 | 1 | 1 | 1 | 1 |

1: Patient demographics are clearly described.

2: Patient’s history is clearly described and presented as a timeline.

3: Current clinical condition of the patient on presentation are described.

4: Diagnostic tests or assessment methods and the results are clearly described.

5: Intervention (s) or treatment procedure(s) are clearly described.

6: Post-intervention clinical condition is clearly described.

7: Adverse events (harms) or unanticipated events identified and described.

8: The case report provides takeaway lessons.


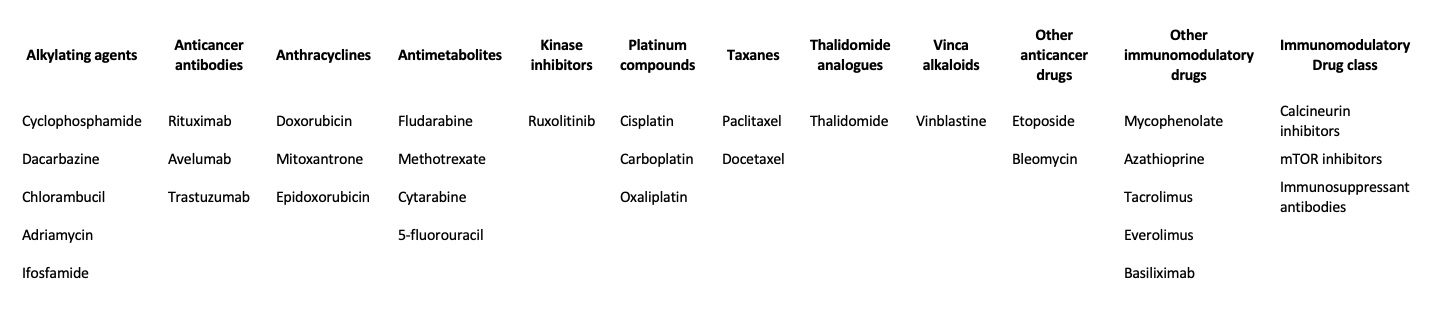
**Appendix D: Anti-cancer agents and drug class**
